# A Study on the Psychological Attributes of Survivors Who Experienced Downsizing in China

**DOI:** 10.3390/ijerph192316071

**Published:** 2022-12-01

**Authors:** Byung Hee Lee, Li Cai, Jing Liu, Yu Jin Chang

**Affiliations:** 1 School of Business, Hanyang University, Seoul 04763, Republic of Korea; 2 Haile College of Business, Northern Kentucky University, Highland Heights, KY 41099, USA; 3 Division of Ari College of Liberal Arts, Anyang University, Anyang 14028, Republic of Korea

**Keywords:** downsizing, perceived justice, psychological contract violation, affective commitment, turnover intention

## Abstract

This study aims to examine how perceived justice affects downsizing survivors’ attitudes, from the psychological contract perspective. By using data collected through surveys from employees of the Industrial and Commercial Bank of China (ICBC) who have recently survived layoffs, we examine the relationships between perceived justice, the survivors’ psychological status, and their attitudes after downsizing. The hypothesis was verified through path analysis using SPSS 26.0 and Amos 23.0. Our findings are as follows. Perceived justice has a negative effect on psychological contract violation and a positive effect on trust. Psychological contract violation influences affective commitment negatively and influences turnover intention positively. Trust is positively related to affective commitment and negatively related to turnover intention. We hope that this study will be a useful piece of data that can provide guidelines for inducing positive behavior of members in downsized organizations.

## 1. Introduction

In 2018, many Chinese firms experienced a harsh economic winter and the wave of consequent layoffs became a big social issue in China. Not only were there a large number of layoffs in private enterprises, but state-owned enterprises (SOEs) also downsized substantially. Because working in the SOEs has long been regarded as having the so-called “iron rice bowl” which means a stable, lifelong job [1], this extensive downsizing has attracted a lot of attention. According to the banks’ financial reports, up to the end of June 2018, the number of employees in China’s “Big Four” state-owned commercial banks decreased by 26,000, compared with the end of 2017. The number of employees in the four major banks has also dropped by as much as 70,000 since 2015. With the rapid development of internet technology and artificial intelligence, this may just be the beginning. Downsizing is expected to occur more actively due to the spread and popularization of internet banking and mobile banking.

Downsizing, which is defined as “a purposeful reduction in the size of an organization’s workforce” [2,3], has been extensively studied. Compared with numerous studies focused on laid-off workers, scholars have found a lack of research on downsizing survivors who remain at work after downsizing, and have started to pay great attention to the survivors because they are the ones who determine the future development and success of the organization [4,5]. Studies have demonstrated that as well as the laid-off workers, the survivors are also profoundly affected by downsizing [3,6,7]. Downsizing can make survivors experience “survivor syndrome”, which means the “mixed bag of behaviors and emotions often exhibited by remaining employees following an organizational downsizing” [8]. Downsizing can also cause survivors to have complex mental states and behavioral tendencies, such as feeling stressed, anxious, depressed; having low commitment; or intending to leave the job because of the increased workloads, the changed tasks, the risk of job loss in the future, and so on [9,10].

Existent studies show that how fair the downsizing is perceived by survivors to be influences the changes in their psychological status and attitudes, and scholars have stressed the importance of justice in the downsizing process [6,11,12]. The psychological mechanism behind the relation between perceived justice and downsizing survivors’ attitudes is still under-researched. The relationship between employees and their organization can be defined as a psychological contract, which is an unwritten agreement about perceived mutual obligations between them [13]. The content of the psychological contract has been found to be crucial in predicting employee’s attitudes and behaviors [14,15]. When an organization breaches the psychological contract with an employee, the employee’s perceived obligations toward the organization will decrease, resulting in negative attitudes, such as decreased commitment [5]. Downsizing has been found to break the balance of the psychological contract between employees and their organization [16], resulting in changes in attitudes and behaviors following the downsizing as the perceptions of the psychological contract change. As such, in this study we explore the mechanisms underlying the relationship between perceived justice and survivors’ attitudes from the psychological contract perspective. Specifically, we examine the relationships among perceived justice, psychological contract violation and trust, and affective commitment and turnover intention.

Downsizing, defined as the intentional reduction in the size of an organization’s workforce, has been extensively studied [2,3]. However, compared to numerous studies focusing on laid-off workers, there is a paucity of research on survivors who remain after downsizing [4,5]. Survivors are the key players who determine the future development and success of an organization, so attention should be paid to research related to survivors. This study attempts to fill the research gap by focusing on survivors who survived after downsizing. So far, most studies on downsizing have been carried out by assessing samples from Western countries. In addition, research on turnover intention started late in China because of the planned economy in the past. With the deepening of economic system reform and the establishment of a socialist market economic system, scholars did not concentrate on turnover intention until the 1990s. The study of survivors’ turnover intention following downsizing in Chinese organizations is still insufficient.

This study, therefore, reveals the downsizing survivors’ psychological process from perceived justice to their affective commitment and turnover intention, and confirms the importance of justice during downsizing. Given that extensive downsizing has occurred frequently in China in recent years and that many experts predict that this phenomenon will continue in the next few years, this study could provide some insights and recommendations for Chinese firms. To ensure the stable and long-term development of the firm, managers need to create appropriate and fair strategies to minimize the negative impacts downsizing might bring, and pay more attention to the survivors’ attitudes and reactions.

We hope this study provides decision-makers and managers with guidance and suggestions when they implement decisions in the process of downsizing, and reminds them of the importance of fairness in downsizing.

## 2. Literature Review and Hypotheses

### 2.1. Organizational Downsizing

Organizational downsizing, short for downsizing, which can also be called layoff, redundancy, etc., refers to “a purposeful reduction in the size of an organization’s workforce” [2,3]. Organizational downsizing has generated lots of attention among scholars and managers. Extant research states that firms might downsize because of economic depression or market competition [17], or might use downsizing as a strategy to improve efficiency, profitability, and competitiveness [2,18]. As for consequences, downsizing has also been found to cause negative impacts on the organization and individual employees—not only those who lost jobs, but also those who survived the downsizing [3,6,7]. Due to workforce reductions, employees’ stress rises, and the organization and individuals are required to cope and adapt to new situations [19].

Survivors, who are the remaining employees after downsizing, could experience “survivor syndrome”, which is a new psychosocial problem that downsizing brings about [8,20]. Noer [9] presented 12 different kinds of negative emotions that survivors may experience after downsizing. Kivimäki et al. [10] indicate that downsizing is associated with increased levels of workloads and job insecurity, and thus decreased levels of skill discretion and participation. Meanwhile, research has also shown that the negative impacts of downsizing can be reduced, to a certain extent, through a fair downsizing process and effective communication [12]. Downsizing makes survivors realize the uncertainty of their jobs, and the risk of job loss in the future causes them to have more concern about whether the procedure or process of downsizing is fair or not [10,12]. If they perceive any unfairness about downsizing, it can lead to negative psychological emotions and attitudes toward their work and organization [5,6]. Leung and Chang [21] investigated changes in four kinds of psychological status (i.e., job stressors, affective commitment, continuance commitment, and job security), and argued that these psychological impacts act as mediators in the relationships between downsizing, perceived justice, survivor syndrome, work effort, and turnover intention.

### 2.2. Perceived Justice

Organizational justice is a concept regarding employee perceptions of fairness in the workplace [22]. It is beneficial to the cultivation of loyalty and trust from employees and can improve the positive attitudes of employees [23]. According to Greenberg [24], there are three reasons why a sense of fairness is important in an organization. Firstly, if supervisors implement decisions fairly, employees are more committed to the organization and thus have better performance [25]. Secondly, the perception of justice can improve cooperation among employees, because it can make people engage in their groups and build a sense of community and belonging [24]. Lastly, employees feel respected if they receive fair treatments [24]. All of these can lead the organization’s operation into a virtuous circle.

Organizational justice has dimensions of distributive justice and procedural justice [22,26]. Distributive justice is the fairness about the allocation or distribution of rewards or resources and is more outcome-oriented [27]. Procedural justice refers to the fairness of the process during resource allocation and distribution decision-making [28]. This dimension is related to the extent to which employees have a voice and express their views in the decision-making procedure. It involves the transparency of the processes and the consistency of the decision’s implementation [22].

Prior studies indicate that distributive justice is not related to organizational commitment when procedural justice is controlled for [11,29]. Rather, procedural justice explains the majority of the variances in organizational commitment and trust [30,31]. The perception of procedural justice has been found to influence attitudes about the organization, resulting in changes in employees’ behaviors [32]. In addition, existing research has stressed the role of procedural justice on psychological status during organizational restructuring [6,11]. Thus, in this paper, we focus on procedural justice and investigate the psychological impacts of procedural justice that survivors perceived during downsizing.

### 2.3. Psychological Contract Violation

Scholars state that downsizing or restructuring can break the balance of psychological contract between employees and their organization [16]. The psychological contract refers to the perceived mutual obligations in the relationship between employees and employers in an organization [13]. It is an intangible agreement about the content of the social exchange between employees and the organization [33]. Psychological contract can influence how people feel and behave, and is highly valued because it can improve employees’ loyalty and performance [34,35,36].

According to the social exchange theory and the norm of reciprocity, employees perceive their inputs as the contribution to their organization and expect the organization to show corresponding responsibilities and promises [37,38]. When the organization fails to fulfill an expected promise, it will result in a break of the balance of the psychological contract, which then may lead to psychological contract violation [39].

Psychological contract violation (PCV) is the “feelings of anger and betrayal that are often experienced when an employee believes that the organization has failed to fulfill one or more of those obligations” [39]. It is strongly related to the emotional experience of individuals.

Employees will have negative feelings such as distrust and anger due to psychological contract violation, which in turn influence employees’ behaviors and responses [40]. Turnley and Feldman [16] proposed a framework, namely the exit, voice, loyalty, and neglect (EVLN) typology, for understanding employees’ responses to psychological contract violation. In response to psychological contract violation, employees may decrease the number of extra-role or organizational citizenship behaviors they engage in and put in less effort and attention [41]. In addition, there will be increased exiting of employees, which means the employees are more likely to leave the firm.

Employees expect to be treated fairly in the downsizing process. When they do not receive desired outcomes and perceive the process as unfair, the balance of psychological contract in their mind will be broken [40]. The injustice of the procedure will arouse intense discontent and negative feelings among employees, and the organization will be viewed as the breaker of the psychological contract because they cannot fulfill their obligation [6]. Although there is not a formal contract about the fairness of the procedure, employees tend to believe there is an invisible psychological contract the organization should obey. The employees’ perception of procedural fairness reduces the likelihood of psychological contract violation [6]. When employees experience fair treatment from their organization, it further reinforces their psychological contract. Therefore, the first hypothesis is proposed as follows.

**H1:**  *Perceived justice is negatively related to psychological contract violation*.

### 2.4. Trust

Trust refers to “one’s expectations, assumptions, or beliefs about the likelihood that another’s future actions will be beneficial, favorable, or at least not detrimental to one’s interest” [42]. Trust plays an important role in work outcomes and the process of performance appraisal. Trust in the organization is a key variable to predict employees’ attitudes and behaviors. Empirical evidence shows that there is a link between organizational trust and organizational commitment, intention to leave, and citizenship behavior [42,43]. Furthermore, trust is closely related to the perception of organizational justice. Researchers indicate that when employees perceive the procedure as fair, they will show a higher level of trust in the organization [44,45]. However, the failure to fulfill fairness as the obligation employees expect from the organization causes the breach of the psychological contract, resulting in the decline of employees’ trust in the organization [5]. On the contrary, if the survivors perceive the organizational downsizing is fair enough, it is not likely to affect the balance of the psychological contract and their loyalty to the organization. Moreover, the survivors may feel recognized by the organization, which in turn increases their trust in the organization. Thus, we propose:

**H2:**  *Perceived justice is positively related to trust*.

### 2.5. Affective Commitment

Organizational commitment can be viewed as the psychological attachment of an individual to their organization. It is a widely used predictor of employees’ behaviors, such as job performance and turnover [46]. The success of an organization depends quite as much upon employees’ commitment towards their jobs. Allen and Meyer [47] divide organizational commitment into three dimensions: affective, continuance, and normative commitment. They define affective commitment as the emotional attachment to, identification with, and involvement in the organization. Continuance commitment refers to commitment engendered based on the costs that employees associate with leaving the organization, and normative commitment reflects employees’ feelings of obligation to remain with the organization [47]. While those with strong continuance commitment think they need to stay due to costs of turnover and those with strong normative commitment feel they ought to stay because of social morality, employees with strong affective commitment stay in the organization because they want to [47,48,49].

Affective commitment is the most desirable dimension of organizational commitment, and organizations are more eager to enhance this form of commitment in their employees. Employees with high affective commitment display an unwillingness to leave because they feel comfortable in the relationship with their organization [47]. When employees have strong affective commitments, they are more motivated and devote more effort to their work, and thus exhibit better work performance and organizational citizenship behavior [50]. For the above reasons, we focus on the affective commitment of survivors.

Downsizing changes the relationships among the coworkers and the organizational environment as well. It leads to increased workload and changes of work contents for survivors because they have to deal with the work that was previously managed by the laid-off workers [51]. Survivors may resist these changes and pressures by withdrawing or lessening their commitment to their organization [4].

According to social exchange theory and the norm of reciprocity, when employees perceive that the organization fails to meet the obligations toward them as they expect, they will reduce their obligations toward the organization to balance the relationship with their organization again [38,52]. Therefore, if downsizing causes the imbalance of a psychological contract between employees and their organization, which leads to psychological contract violation, employees might lose motivation in their workplace and reduce their inputs and work performance.

Extant studies indicate that organizational trust can reduce perceptions of threat stemming from downsizing and facilitate more constructive survivor attitudes [19]. In contrast, survivors will be less committed to their work and show negative emotions in their workplace if downsizing results in the loss of trust.

Based on that mentioned above, two hypotheses are developed as follows.

**H3a:**  *Psychological contract violation is negatively related to affective commitment*.

**H4a:**  *Trust is positively related to affective commitment*.

### 2.6. Turnover Intention

Turnover intention is workers’ intentions, desires, and plans to quit their job, and is a significant and important predictor of turnover [53,54]. Because high turnover rates can lead to irretrievable losses for the organization [55], firms pay much attention to the reasons and factors that influence employees’ turnover and turnover intention, and take measures to avoid this situation.

Scholars have studied individual and organizational factors that affect employees’ turnover intention. Individual factors include employees’ attitudes toward their work and organization [56,57]. Organizational factors include organizational justice, the relationship with supervisors, and so forth. Organizational justice is significantly related to turnover intention, and perceived organizational support mediates their effects on organizational commitment and intention to leave [58]. Employees will show better work attitudes and performance and lower intention to leave if they have a high degree of satisfaction with their supervisors.

Scholars have found that one of the forms that employees respond to psychological contract violation is increased turnover [41]. As turnover intention is the next logical step after experienced dissatisfaction in the withdrawal process [59], the violation of the psychological contract caused by downsizing is likely to result in a high level of turnover intention [6].

Previous research has demonstrated that if employees do not trust their organization, for example if they do not believe that their organization is concerned about their interests and do not think they can receive what they expect from their firm, they might feel threatened and insecure about their future work in the organization and are likely to show less commitment and even withdrawal behaviors [3,5]. Conversely, organizational trust tends to facilitate commitment, which in turn decreases the turnover intention. Based on those mentioned above, the hypotheses are proposed as follows.

**H3b:**  *Psychological contract violation is positively related to turnover intention.*

**H4b:**  *Trust is negatively related to turnover intention.*

**Figure 1:**
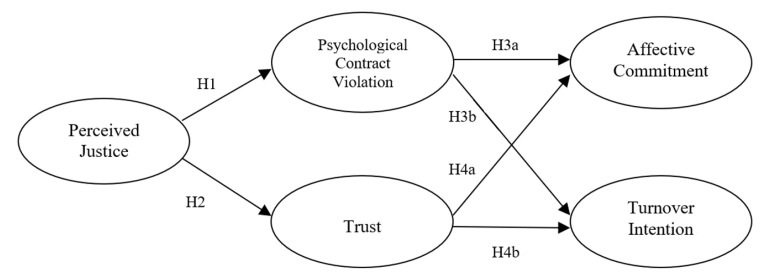
Research model.

Figure 1 illustrates the hypothesized relationships in our study.

## 3. Research Methodology

### 3.1. Sample and Data Collection

The data was collected from the employees who had just survived the downsizing of Industrial and Commercial Bank of China (ICBC), a state-owned bank. The survey was conducted online and a total of 302 responses were received.

A total of 11 of the respondents said that they had not experienced the downsizing at ICBC because they were hired after that period. Thus, only the responses of the remaining 291 respondents, who answered that they had experienced downsizing at ICBC, were used for analysis. Although 291 questionnaires were collected, 14 respondents chose the same answer for each item. Therefore, data from these respondents were replaced by the median of each item. The sample characteristics are shown in Table 1.

### 3.2. Measures

As shown in Table 2, the questionnaire consisted of 33 items for measuring the five variables: perceived justice, psychological contract violation, trust, affective commitment, and turnover intention, and all items are developed with a 7-point Likert-type scale. Respondents were asked to answer their thoughts and feelings about all items (1 = strongly disagree; 7 = strongly agree).

## 4. Results

### 4.1. Reliability of Questionnaire

According to the initial reliability analysis, Cronbach’s alpha of trust and affective commitment are lower than 0.7, and the CITC of TR3, TR5, TR7, and AC4 are lower than 0.3. After deleting TR5, which has the lowest CITC among all items of trust, Cronbach’s alpha of trust (0.708) is above 0.7. However, the CITC of TR3 (0.190) and TR7 (0.184) are still less than 0.3. After deleting TR7, the CITC of TR3 is 0.056. Thus, TR3 is deleted as well. Cronbach’s alpha of trust is 0.848, which is higher than 0.7, and the remaining items’ CITC are all higher than 0.3. As for affective commitment, after deleting AC4, Cronbach’s alpha is 0.738 and the rest of items’ CITC are above 0.3. Reliability of the modified scale is shown in Table 3 and the variables were verified for their reliability.

### 4.2. Exploratory Factor Analysis

The KMO to judge the adequacy of the sample was 0.873, and the result of Bartlett’s sphericity test was statistically significant, and factor analysis was performed. As to factor analysis, principal component analysis was used as an extraction method, and varimax was selected as a rotation method. The final results of factor analysis are shown in Table 4.

Lastly, considering that the same data source, the same measurement environment, and the same questionnaire might lead to covariation between predictor variables and criterion variables, the common method bias was checked by Harmons’ single-factor test [62]. In this test, the method of principal axis factoring was used to extract only one factor. The result showed that a single factor extracted 26.772% of total variance, which was far less than 50%. Therefore, the threat of common method bias is minimal.

### 4.3. Confirmatory Factor Analysis

Confirmatory factor analysis (CFA) was conducted to assess the fit of the measurement model before specifying the hypothesized relationships among latent variables because the measurement model specifies the relationships between latent variables and their respective indicators, and it makes little sense to build the structural model if the indicators fail to measure the latent variables [7,63].

Firstly, according to the result of model fit in the initial CFA (IFI = 0.906, TLI = 0.893, CFI = 0.906, RMSEA = 0.080) and modification indices, PCV1, PCV3, and PCV4 were removed due to model fit discrepancies. After the deletion of these three items, the fit of the measurement model was concluded, as in Table 5.

Secondly, reliability and convergent and discriminant validity were checked in Amos and can be seen in Table 6. According to the result of the validity check shown in Table 6, all values of CR (composite reliability) are above 0.7, values of AVE (average variance extracted) are above 0.5, and values of square root of AVE are greater than correlations. Thus, there are no validity concerns about this model and it can be perceived that the measurement model is decent.

### 4.4. Structural Model

Before examining the structural model, the bivariate Pearson correlation was conducted to assess relationships among variables. The results are shown in Table 7.

**Figure 2:**
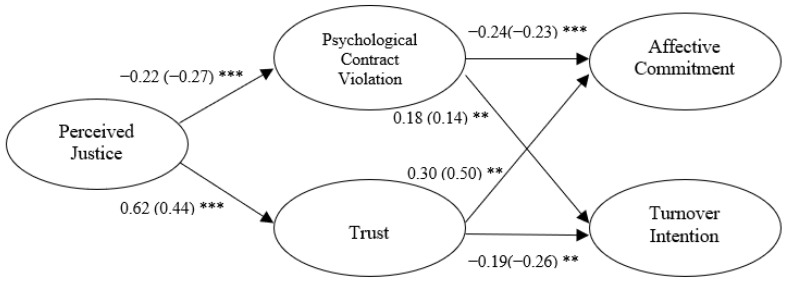
Hypothesis testing results. Notes: ** *p* < 0.01, *** *p* < 0.001. Unstandardized path coefficients are shown in parentheses.

A structural model was built to examine the relationships among latent variables. Gender, marital status, and previous downsizing experience were used as control variables in the model. According to the result of modification indices, the error terms of two dependent variables were connected because the M.I. (43.591) between them were high. After running the model again, the model fit was satisfactory (CMIN = 472.97, df = 253, CMIN/df = 1.869, *p* < 0.001, IFI = 0.949, TLI = 0.938, CFI = 0.948, RMSEA = 0.055). The results of path analysis are concluded in Figure 2 below.

Figure 2 shows that the coefficient of the path between perceived justice and psychological contract violation is −0.22 with a *p*-value less than 0.001, which means that the influence of perceived justice on psychological contract violation is significant and negative. Thus, H1 is supported.

The coefficient of the path between perceived justice and trust is 0.62 and *p*-value is smaller than 0.001. Perceived justice has a positive and significant effect on trust, and therefore H2 is supported.

The direct relationship between psychological contract violation and affective commitment is significant (*p* < 0.001) and the coefficient is −0.24. Thus, psychological contract violation is negatively related to affective commitment, and H3a is supported. Psychological contract violation significantly influences turnover intention in a positive way because the coefficient of the path is 0.18 and *p*-value is less than 0.01. Hence, H3b is supported.

The coefficient of the path between trust and affective commitment is 0.30 and *p*-value is smaller than 0.001, which means that trust is positively related to affective commitment. Thus, H4a is supported. Trust has a negative influence on turnover intention, because the coefficient is −0.19, and it is significant with *p*-value less than 0.01. Therefore, H4b is also supported.

## 5. Discussion and Conclusions

This study was motivated to fill a gap in the downsizing literature: there is a relative lack of research related to downsizing survivors compared to laid-off workers who have gone through downsizing. We aimed to verify the relationship between perceived justice and attitudes of downsizing survivors from a psychological contract perspective in an organization experiencing downsizing. Specifically, we tested the relationships among perceived justice, psychological contract violation, trust, affective commitment, and turnover intention through data collected from the employees who survived downsizing at ICBC in the same year. The empirical findings show that perceived justice has a negative effect on psychological contract violation and a positive effect on trust. Psychological contract violation influences affective commitment negatively and influences turnover intention positively. Trust is positively related to affective commitment and negatively related to turnover intention. This means that when an organization member recognizes unfairness in an employment relationship, a psychological contract violation occurs, and trust in the organization decreases, which negatively affects the immersion of the individual object. These results are in agreement with previous studies [3,5,6,19]. Recognition of psychological breach of contract not only reduces organizational members’ trust in superiors or organizations, but also can cause anti-industrial task behavior, which can be a factor in reducing organizational effectiveness. It will be important to minimize the psychological contract violation awareness that organizational members may have. This study will contribute to strengthening the theory or confirming its feasibility by empirically verifying the psychological contract theory, almost for the first time in survivors who have gone through downsizing, and it is expected to trigger many follow-up studies.

The findings offer some implications as follows. Firstly, it confirmed the significance of perceived justice toward downsizing, which is consistent with the previous studies [6,11,12]. Employees’ perceptions of downsizing procedural justice can reduce the possibility of psychological contract violation and increase the level of trust toward the organization among survivors. Thus, survivors will be more committed to their organization and less willing to leave. As the negative impacts of perceived injustice about the downsizing on survivors’ psychological contracts, attitudes, and behavioral reactions can be significant and last for a long time, employers should pay attention to fairness when implementing downsizing. A climate of fairness is important in a company, especially during the specific period of downsizing. It is significant to take steps to maintain fairness and emphasize the importance of perceived fairness in the process of downsizing. Employers should care about the interests of their employees and apply job decisions consistently, instead of only going after the profit of the firm.

Secondly, this study contributes to revealing the downsizing survivors’ psychological process from perceived justice to their affective commitment and turnover intention. Employees expect to be treated fairly during downsizing. When the organization fails to meet their expectations, the employees may think that the psychological contract between them and the organization is violated and this may reduce trust toward the organization. Then they tend to reduce the level of commitment toward the organization and the turnover intention also tends to increase to rebalance the relationship between them and the organization. Organizational decisions have a profound effect on employees’ psychological contracts with the organization. Thus, if the decision keeps the organization from fulfilling mutually unwritten obligations, the firm needs to notice the situation and provide accurate and complete information to employees. It helps to reach the goal of maintaining the psychological contract and trust if employees can understand the reasons for inevitable unfair decisions. In addition, employers can renegotiate the psychological contract with employees, and make appropriate strategies which can make up for losses to avoid the perception of psychological contract breach and negative attitudes or reactions of survivors [6].

At the same time, there are several limitations in this study. Firstly, this study focuses on only one specific organization—ICBC, and thus, due to limited generalizability, it is necessary to undertake further research on different organizations. Actually, a lot of companies have experienced downsizing in recent years in China. It might be interesting to compare survivors’ attitudes and reactions in different organizations. Secondly, this research only studies downsizing survivors’ attitudes through stressing the psychological process and the significance of perceived justice. However, many other significant aspects are worth paying attention to; for instance, the importance of cultural values and the changes in the work environment.

## Figures and Tables

**Table 1:** Sample characteristics.

Variables	Category	Number (Total = 291)	Percentage (%)
Gender	Male	133	45.7
	Female	158	54.3
Age	20 or younger	5	1.73
	21–30	188	64.6
	31–40	42	14.43
	41–50	31	10.65
	51 or older	25	8.59
Education Level	A high school graduate or lower	17	5.84
	Associate degree	58	19.93
	Bachelor Degree	182	62.54
	Master’s degree or higher	34	11.68
Marital Status	Single	177	60.82
	Married	114	39.18
Numbers of Previous Experienced Downsizing	0	175	60.14
	1	76	26.12
	2	22	7.56
	3	10	3.44
	4 or more	8	2.74
Tenure with Organization	Less than 1 year	72	24.74
	1–2 years	82	28.18
	3–5 years	57	19.59
	6–10 years	13	4.47
	11 years or more	67	23.02

**Table 2:** Variable measurements.

Variables		Items	References
Perceived Justice	PJ1	Job decisions are made by the general manager in an unbiased manner.	[60]
	PJ2	My general manager makes sure that all employee concerns are heard before job decisions are made.	
	PJ3	To make job decisions, my general manager collects accurate and complete information.	
	PJ4	My general manager clarifies decisions and provides additional information when requested by employees.	
	PJ5	All job decisions are applied consistently across all affected employees.	
	PJ6	Employees are allowed to challenge or appeal job decisions made by the general manager.	
Psychological Contract Violation	PCV1	You felt betrayed with regard to the downsizing implemented by your organization.	[39]
	PCV2	You felt angry with regard to the downsizing implemented by your organization.	
	PCV3	You felt resentful with regard to the downsizing implemented by your organization.	
	PCV4	You felt shock with regard to the downsizing implemented by your organization.	
	PCV5	You felt insecure with regard to the downsizing implemented by your organization.	
	PCV6	You felt a loss of trust with regard to the downsizing implemented by your organization.	
	PCV7	You felt unfair with regard to the downsizing implemented by your organization.	
	PCV8	You felt disappointed with regard to the downsizing implemented by your organization.	
Trust	TR1	I believe my employer has high integrity.	[42]
	TR2	I can expect my employer to treat me in a consistent and predictable fashion.	
	TR3	My employer is not always honest and truthful.	
	TR4	In general, I believe my employer’s motives and intentions are good.	
	TR5	I don’t think my employer treats me fairly.	
	TR6	My employer is open and upfront with me.	
	TR7	I am not sure I fully trust my employer.	
Affective Commitment	AC1	I would be very happy to spend the rest of my career with this organization.	[47]
	AC2	I enjoy discussing my organization with people outside it.	
	AC3	I really feel as if this organization’s problems are my own.	
	AC4	I think that I could easily become as attached to another organization as I am to this one.	
	AC5	I do not feel like ‘part of the family’ at my organization.	
	AC6	I do not feel ‘emotionally attached’ to this organization.	
	AC7	This organization has a great deal of personal meaning for me.	
	AC8	I do not feel a strong sense of belonging to my organization.	
Turnover Intention	TI1	I intend to leave the organization in the near future.	[61]
	TI2	In the last few months, I have seriously thought about looking for a new job.	
	TI3	Presently, I am actively searching for another job.	
	TI4	I often think about quitting my job.	

**Table 3:** Reliability of the scale.

Variables	Items	CITC	Cronbach’s Alpha
Perceived Justice	PJ1	0.648	0.913
	PJ2	0.824	
	PJ3	0.786	
	PJ4	0.811	
	PJ5	0.761	
	PJ6	0.710	
Psychological Contract Violation	PCV1	0.719	0.946
	PCV2	0.846	
	PCV3	0.822	
	PCV4	0.773	
	PCV5	0.717	
	PCV6	0.850	
	PCV7	0.864	
	PCV8	0.825	
Trust	TR1	0.763	0.848
	TR2	0.739	
	TR4	0.786	
	TR6	0.759	
Affective Commitment	AC1	0.462	0.738
	AC2	0.371	
	AC3	0.541	
	AC5	0.370	
	AC6	0.455	
	AC7	0.448	
	AC8	0.524	
Turnover Intention	TI1	0.888	0.879
	TI2	0.892	
	TI3	0.859	
	TI4	0.640	

**Table 4:** Factor analysis.

Variables	Items	Rotated Component Matrix
Perceived Justice	PJ1	0.725
	PJ2	0.843
	PJ3	0.840
	PJ4	0.853
	PJ5	0.815
	PJ6	0.759
Psychological Contract Violation	PCV1	0.765
	PCV2	0.872
	PCV3	0.854
	PCV4	0.831
	PCV5	0.776
	PCV6	0.883
	PCV7	0.900
	PCV8	0.860
Trust	TR1	0.763
	TR2	0.739
	TR4	0.786
	TR6	0.759
Affective Commitment	AC5	0.760
	AC6	0.835
	AC8	0.795
Turnover Intention	TI1	0.888
	TI2	0.892
	TI3	0.859
	TI4	0.640
KMO	0.873	
Sig.	0.000	

**Table 5:** Model fit of the measurement model.

Model	IFI	TLI	CFI	RMSEA
Default Model	0.950	0.941	0.950	0.060

**Table 6:** Validity and reliability.

	CR	AVE	MSV	MaxR(H)	AC	PJ	PCV	TR	TI
AC	0.802	0.576	0.284	0.823	0.759				
PJ	0.914	0.642	0.377	0.924	0.182	0.801			
PCV	0.932	0.735	0.055	0.943	−0.234	−0.232	0.857		
TR	0.850	0.592	0.377	0.905	0.333	0.614	−0.041	0.770	
TI	0.883	0.655	0.284	0.897	−0.533	−0.117	0.134	−0.242	0.809

Notes: AC = Affective Commitment, PJ = Perceived Justice, PCV = Psychological Contract Violation, TR = Trust, TI = Turnover Intention.

**Table 7:** Correlation.

	PJ	PCV	TR	AC	TI
PJ	1				
PCV	−0.213 **	1			
TR	0.549 **	−0.041	1		
AC	0.149 *	−0.226 **	0.253 **	1	
TI	−0.131 *	0.145 *	−0.222 **	−0.482 **	1

Notes: * *p* < 0.05, ** *p* < 0.01, PJ = Perceived Justice, PCV = Psychological Contract Violation, TR = Trust, AC = Affective Commitment, TI = Turnover Intention.

## Data Availability

Not applicable.
